# Association of Hospital Incentive Care Management Partnerships for Uninsured Patients With Emergency Department Utilization

**DOI:** 10.1001/jamanetworkopen.2023.22798

**Published:** 2023-07-11

**Authors:** Sarah Gareau, Ana López-De Fede, Zhimin Chen, Nathaniel Bell, Kathy Mayfield-Smith

**Affiliations:** 1 Institute for Families in Society, University of South Carolina, Columbia; 2 College of Nursing, University of South Carolina, Columbia

## Abstract

**Importance:**

The South Carolina (SC) Healthy Outcomes Plan (HOP) program aimed to expand access to health care to individuals without insurance; it remains unknown whether there is an association between the SC HOP program and emergency department (ED) use among patients with high health care costs and needs.

**Objectives:**

To determine whether participation in the SC HOP was associated with reduced ED utilization among uninsured participants.

**Design, Setting, and Participants:**

This retrospective cohort study included 11 684 HOP participants (ages 18-64 years) with at least 18 months of continuous enrollment. Generalized estimating equations and segmented regression of interrupted time-series analyses of ED visits and charges were conducted from October 1, 2012, to March 31, 2020.

**Exposures:**

Time intervals related to the HOP were 1 year before and 3 years after participation.

**Main Outcomes and Measures:**

ED visits per 100 participants per month and ED charges per participant per month overall and by subcategory.

**Results:**

The mean (SD) age of the 11 684 participants in the study was 45.2 (10.9) years; 6293 (54.5%) were women; 5028 (48.4%) were Black participants and 5189 (50.0%) were White participants. Over the study period, the mean (SE) number of ED visits decreased by 44.1%, from 48.1 (5.2) to 26.9 (2.8) per 100 participants per month. The mean (SE) ED charges were reduced to $858 ($46) per participant per month, a decrease from a mean (SE) of $1583 ($88) per participant per month 1 year before HOP implementation. There was an immediate level decrease of 40% (relative risk [RR], 0.61; 99.5% CI, 0.48-0.76; *P* < .001) from the preenrollment period, with a sustained reduction trend of 8% (RR 0.92; 99.5% CI, 0.89-0.95; *P* < .001) during the postenrollment period. A level change for ED charges was detected, at a decrease of 40% (RR 0.60; 99.5% CI, 0.47-0.77; *P* < .001) directly after HOP enrollment with a subsequent downward trend of 10% (RR 0.90; 99.5% CI, 0.86-0.93; *P* < .001) for the postenrollment period.

**Conclusions and Relevance:**

In this retrospective cohort study, proportions and charges of ED visits by uninsured patients saw immediate and sustained decreases after HOP enrollment. Reducing ED charges may have been driven by decreasing the ED as the primary point of patient care, especially for high-frequency users. These findings have implications for other nonexpansion states seeking to maximize uninsured compensation for low-income populations through improved outcomes.

## Key Points

**Question:**

Was South Carolina’s Healthy Outcomes Plan (HOP) associated with a change in emergency department (ED) visits for uninsured patients with high health care costs and needs?

**Findings:**

In this cohort study that included 11 684 HOP participants, a statistically significant 3-year 44.1% reduction in ED service use, with an estimated net reduction in ED charges of $725 per participant, was observed.

**Meaning:**

These findings suggest that state-funded hospital-community partnerships that address access to behavioral health care and the safety net, patient activation, and the social determinants of health would benefit other states.

## Teaser

This cohort study evaluates whether participation in South Carolina’s Health Outcomes Plan program was associated with reduced emergency department utilization among uninsured participants with high health care costs and needs.

## Introduction

The South Carolina (SC) Healthy Outcomes Plan (HOP) was implemented in 2012 to expand access to health care services to uninsured individuals. Distinct from previous programs designed to lower acute service use,^1,2,3,4,5^ HOP was funded through a state legislative proviso. It incentivized all hospitals in the state with emergency departments (EDs) to participate. In total, 43 HOP partnerships were developed. As a result of HOP, SC has consistently had lower uninsured rates compared with other Southeastern US states that similarly elected not to expand their Medicaid eligibility thresholds allowed under the Patient Protection and Affordable Care Act.^6^

Several HOP provisions were designed to increase access to health care services, including (1) regional contracts requiring price and quality transparency, (2) medical home provision, (3) full disproportionate share hospital (DSH) compensation to rural hospitals, and (4) additional funding to qualifying safety net hospitals. In exchange, each hospital contracted with selected primary health care and other clinicians to comanage patients with chronic illness, no insurance, and high rates of ED use. We examined trends in ED visits and charges from 2012, when the HOP program came into effect, to 2020. Specifically, we aimed to answer the following question: is HOP associated with significant changes in the number of ED visits and charges by uninsured individuals?

## Methods

### The HOP Program

The HOP program is a novel statewide comprehensive policy intervention model designed for the uninsured and administered through the South Carolina (SC) Department of Health and Human Services (SCDHHS) Medicaid program. Using monetary incentives and quality transparency efforts, the program focuses on patients with chronic illness, high needs, and high use of acute care services to reduce per capita cost of health care as well as improve patient experiences of care. To be eligible for the HOP program, participants must be uninsured adults ages 18 to 64 years who are both below 138% of the federal poverty level (FPL) and high-cost and high-need utilizers, as defined by frequent ED utilization, with at least 2 physical and/or behavioral health needs.

To provide more comprehensive care coordination, the program emphasizes addressing the social determinants of health, patient engagement, and behavioral health needs using annual screenings such as the Social Determinants Assessment, Patient Activation Measure, and the Global Appraisal of Individual Needs–Short Screener; care plans are also required within 60 days of enrollment. Partners, a core component of HOP, play a critical role in ensuring that participants receive needed clinical and nonclinical services. To avoid unnecessary ED visits, partners assist with facilitating self-care and health management by providing primary care and connecting participants with other services. HOPs also encourage the use of primary and specialty care through a variety of patient financial incentive models, including waived copays, free visits, and reduced rates.

Eligibility is verified annually by HOP coordinators employed by the hospital or partners with participant enrollment information reported monthly to SCDHHS. Because the evaluation of HOP requires identifiable records for linkage, each patient signs informed consent forms allowing their enrollment, care plan, and medical claims information to be shared. As part of this larger program evaluation, this study received an institutionally determined exemption from institutional review board approval through the University of South Carolina’s business associate agreement with SCDHHS. This study followed the Strengthening the Reporting of Observational Studies in Epidemiology (STROBE) reporting guideline.

### Study Design and Setting

We conducted a retrospective cohort analysis using Uniform Billing (UB)–04 all-payer hospital billing data for ED discharges (2012-2020) from the state data warehouse at the SC Revenue and Fiscal Affairs Office. Federal fiscal years (FFYs) were used to align with participant enrollment periods (October 1 to September 30). Personal identifiers were used to determine the month of enrollment, enrollment status, and matching criteria for linking with UB-04 records.

### Study Cohort

A total of 31 166 unique HOP participants were enrolled in HOP between FFY 2014 and 2020. Following findings from the Oregon Health Experiment,^7,8^ data analysis was limited to participants with at least 18 months of continuous enrollment (n = 14 312); having at least 1 ED visit or inpatient stay within 12 months prior to their enrollment into HOP (n = 11 715); and aged between 18 and 64 years. This reduced the analytical sample to 11 684 participants (37.5% of total), totaling 193 824 hospital discharge records. Within the cohort, 5608 patients (48.0%) were still actively enrolled; 6076 (52.0%) had been disenrolled as of March 31, 2020, when the sample was pulled. The most common reasons for disenrollment were enrolling in Medicaid or Medicare (1865 [30.7%]), being unable to contact or locate (747 [12.3%]), or having other insurance (680 [11.2%]).

Physical and behavioral health chronic conditions were defined using the Chronic Condition Indicator for the *International Classification of Diseases, Tenth Revision, Clinical Modification* and developed as part of the Healthcare Cost and Utilization Project. Covariates included mean age, age group (18-24, 25-34, 35-44, 45-54, and >55 years), gender (woman or man), self-identified race (White, Black, and other [Hispanic, American Indian, ≥1 race, and unknown]), and rural residence (defined by zip code tabulation area [ZCTA], based on the US Census Bureau 2010 Decennial Census).

### Exposures and Outcomes

The exposure of interest was time since the program’s implementation. For each participant, we considered 12 months from the earliest ED discharge date before the enrollment date as the pre-intervention period (pre-HOP year 0) and 36 months after their enrollment date as the postintervention period (post-HOP years 1-3). ED visits were restricted to those resulting in discharge from the ED and were defined by revenue codes 045X or *Current Procedural Terminology* (*CPT*) codes 99281 to 99285 and standardized as per 100 participants per month. ED charges were standardized as per participant per month and defined using UB charge data; yearly Consumer Price Index of medical services conversion rates were applied to convert all charges into 2020 US dollars. Although some participants were disenrolled prior to 3 years after HOP, their ED visit data for all payers was available for analysis prior to and after enrollment.

We implemented the New York University (NYU) algorithm for classifying ED visits based on acuity. The NYU algorithm uses discharge data to calculate the probability that the event can be classified as nonemergent; emergent, but primary care treatable; emergent, but preventable or avoidable; or emergent, not preventable or avoidable. Utilization level was identified based on ED use prior to enrollment: frequent utilizers (<3 ED visits), high utilizers (3-6 ED visits), and super utilizers (>6 ED visits). Findings from the 2013 Super-Utilizer Summit informed the cutoff points for the classification.^9^ Severity of ED visits prior to enrollment was also identified as limited or minor, low to moderate, moderate, significant threat, and high severity or life-threatening, using *CPT* codes 99281 to 99285. Hospitals were classified as rural-exempt if they met 1 or more of the exemption criteria based on type, ZCTA, persistent poverty, or number of licensed beds.

### Statistical Analysis

Using registration and pre-HOP utilization data, the HOP participant characteristics at the time of enrollment for the intervention were summarized via frequencies and percentages or means and SDs and compared using χ^2^ tests or *t* tests, as appropriate. Interrupted time-series (segmented regression) analysis was used to measure changes in ED visits and charges between the pre-intervention and postintervention periods to account for the absence of a comparison group.^10,11^ To control for possible time-varying effects, ED visits were modeled based on each participant’s pre- and post-HOP enrollment years as an aggregation of individual trends. A sensitivity analysis was conducted to test whether excluding outcome records at the time of HOP registration changed the results and whether the assumptions were held. The model was based on a quasi-likelihood approach with an added scale parameter to adjust the standard errors of the regression estimates. The intervention effects were estimated using generalized estimating equations (GEEs) with Poisson distribution to handle the time-varying and overdispersion of events. The GEE model was also applied to subsequent group analyses. All estimates were adjusted by age, gender, race and ethnicity, and rural residence based on participant characteristics at enrollment. All tests were 2-sided, and *P* < .005 was considered statistically significant to increase the thresholds for detection from large data sets.^12,13^ All statistical analyses were performed in SAS version 9.4 (SAS Institute).

## Results

The mean (SD) age of the 11 684 participants in the study was 45.2 (10.9) years; 6293 (54.5%) were women; 5028 (48.4%) were Black participants and 5189 (50.0%) were White participants. Table 1 presents the study cohort’s demographic characteristics and comorbidities as well as the hospital characteristics of the study cohort and the total HOP participant sample. Among the study cohort, the mean (SD) number of enrollment months was 38.9 (15.1), with 2480 (21.2%) enrolled for 18 to less than 24 months; 3156 (27.0%), 24 to less than 36 months; and 6048 (51.2%), 36 or more months. High and super utilizers accounted for nearly half of all participants in the study cohort. Approximately 30% of the study cohort lived in rural areas, and rural hospitals serviced 13.7% of participants. Most participants had hypertension (8684 [74.3%]) and substance abuse conditions (8772 [72.5%]) during pre-ED visits. Comorbid physical and behavioral health chronic conditions were listed in 40.1% of the participants’ disease profiles (5152 participants), and more than half of their ED visits prior to enrollment were coded as a significant threat or high severity or life-threatening. A comparison of the study sample with the total participant file revealed similar enrollment demographic characteristics for gender, race and ethnicity, and ED utilization severity classification. However, in alignment with the criteria for disenrollment, the analytical cohort was slightly younger (−5.6 percentage points in the 55-64 year age group and 2.4 percentage points in the 35-44 year or 45-54 year age groups) and had higher rates of behavioral health (8.2 percentage points) or comorbid physical and behavioral health conditions (4.4 percentage points).

**Table 1.** Comparison of Demographic Characteristics Between Cohort and All Data

Characteristic	Participants, No. (%)		*P* value
	Study cohort (N = 11 684)	All participants (N = 31 166)	
Age, mean (SD), y	45.2 (10.9)	46.3 (11.4)	<.001
Age group, y			
18-24	632 (5.4)	1065 (5.2)	<.001
25-34	1636 (14.0)	4102 (13.2)	
35-44	2485 (21.3)	5879 (18.9)	
45-54	4468 (38.2)	11 163 (35.8)	
55-64	2463 (21.1)	8407 (26.7)	
Gender			
Women	6293 (54.5)	16 318 (53.1)	.007
Men	5249 (45.5)	14 434 (46.9)	
Race and ethnicity			
White	5189 (50.0)	13 939 (50.8)	.10
Black	5028 (48.4)	12 981 (47.4)	
Other^a^	162 (1.6)	504 (1.8)	
Health conditions			<.001
Diabetes	4797 (41.1)	12 050 (38.7)	<.001
Hypertension	8684 (74.3)	21 983 (70.5)	<.001
CVD	6626 (56.7)	17 043 (54.7)	<.001
Substance abuse	8772 (72.5)	21 098 (67.7)	<.001
Mental health	6385 (54.7)	15 364 (49.3)	<.001
Disease profile			
Chronic condition	2333 (20.0)	6173 (19.8)	<.001
Behavioral health	2783 (23.8)	4855 (15.6)	
Both chronic and behavioral	5152 (40.1)	11 111 (35.7)	
None	1416 (12.1)	9027 (28.9)	
Severity			
Limited or minor	185 (2.2)	449 (2.1)	.75
Low to moderate	540 (6.6)	1709 (7.9)	
Moderate	3008 (36.4)	8114 (37.6)	
Significant threat	2825 (34.2)	7204 (33.4)	
High severity or life-threating	1696 (20.6)	4112 (19.1)	

Abbreviation: CVD, cardiovascular disease.

^a^ Other self-identified races included multiple race (5 participants [3.1%]), American Indian (9 participants [5.6%]), Hispanic (47 participants [29.0%]), and unknown race (101 participants [62.4%]).

By the third year after enrollment, mean (SD) ED charges decreased to $858 ($46) per participant per month (Table 2). This represented a $725 (*P* < .001) net reduction in ED charges, or a 45.8% savings in 2020 dollars. The adjusted mean ED charges for super utilizers showed a reduction of $2853 (*P* < .001) per participant per month, or a 52.6% reduction in total charges from the pre-intervention period. These charges were reflective of a stepwise decrease in the total number of ED visits (Table 2), which had a relative reduction of 44.1% by the end of the third year. As expected, the largest reduction in ED visits was seen among super utilizers, falling from a pre-intervention adjusted mean (SE) of 167.8 (18.4) to 77.4 (8.2) per 100 participants per month over the 3-year period. Percentage ED visits and charges decreases were all significant when stratified by health condition, hospital location, and ED admission type.

**Table 2.** Mean Changes Over 3 Years

Mean metric^a^	Mean (SE) by intervention year				*P* value, pre-intervention vs year 3^b^
	Pre- intervention	Year 1	Year 2	Year 3	
**Change in ED visit utilization, per 100 participants per month **					
ED visits					
Full cohort	48.1 (5.2)	37.7 (3.9)	31.3 (3.2)	26.9 (2.8)	<.001
By type of utilizers					
Super utilizers	167.8 (18.4)	119.6 (12.8)	90.4 (9.2)	77.4 (8.2)	<.001
High utilizers	64.2 (6.1)	47.2 (4.6)	42.0 (4.2)	35.7 (3.6)	<.001
Frequent utilizers	24.2 (2.1)	24.1 (2.2)	21.7 (2.0)	19.0 (1.8)	<.001
By hospital locations					
Rural hospital	33.1 (3.6)	24.7 (2.8)	22.3 (2.6)	21.7 (2.8)	<.001
Urban hospital	51.3 (5.6)	40.4 (4.2)	33.3 (3.4)	28.2 (2.9)	<.001
By health conditions					
Chronic and behavioral	62.9 (6.6)	49.5 (5.3)	40.9 (4.4)	35.1 (3.8)	<.001
Behavioral	56.5 (6.0)	44.5 (4.8)	35.0 (3.8)	29.8 (3.3)	<.001
Chronic	37.5 (5.2)	27.7 (3.4)	25.0 (2.7)	22.4 (2.4)	<.001
None	34.3 (3.6)	28.6 (3.1)	24.4 (2.7)	20.7 (2.3)	<.001
By NYU categories					
Preventable	4.1 (0.5)	3.0 (0.3)	2.7 (0.3)	2.3 (0.3)	<.001
Nonpreventable or avoidable	6.4 (0.4)	5.2 (0.3)	4.3 (0.3)	3.8 (0.2)	<.001
Primary care treatable	10.2 (0.6)	7.9 (0.5)	6.6 (0.4)	5.9 (0.4)	<.001
Nonemergent	9.9 (1.1)	7.5 (0.8)	6 (0.6)	5.1 (0.6)	<.001
**Change in ED visit charges per participant per month, $**					
ED charges					
Full cohort	1583 (88)	1284 (70)	1061 (57)	858 (46)	<.001
By type of utilizers					
Super utilizers	5428 (329)	4018 (251)	3101 (204)	2575 (178)	<.001
High utilizers	2263 (118)	1760 (103)	1492 (97)	1151 (78)	<.001
Frequent utilizers	1000 (52)	932 (53)	835 (50)	653 (44)	<.001
By hospital locations					
Rural hospital	1008 (74)	786 (63)	772 (76)	632 (62)	<.001
Urban hospital	1835 (113)	1518 (93)	1231 (77)	982 (65)	<.001
By health conditions					
Chronic and behavioral	2419 (154)	1963 (131)	1622 (114)	1312 (98)	<.001
Behavioral	1823 (128)	1553 (110)	1177 (92)	911 (75)	<.001
Chronic	1343 (107)	1021 (86)	915 (75)	760 (63)	<.001
None	1000 (74)	980 (86)	847 (78)	635 (87)	<.001
By NYU categories					
Preventable	145 (13)	119 (12)	102 (9)	81 (8)	<.001
Nonpreventable or avoidable	361 (22)	300 (19)	237 (16)	193 (13)	<.001
Primary care treatable	397 (23)	320 (19)	270 (17)	212 (13)	<.001
Nonemergent	291 (24)	226 (18)	186 (15)	149 (12)	<.001

^a^ The generalized estimating equation method estimates the mean, accounting for within-participants correlation across time.

^b^ All models adjusted by age, gender, race and ethnicity, and residence. Statistical significance was set at α = .005.

The overall magnitude of change in ED admissions (Table 3) was a 40% decrease (RR, 0.61; 99.5% CI, 0.48-0.76; *P* < .001), with the level change maintaining a similar trend over the 3 years. The overall effect size (slope) was an 8% decrease in ED visits (RR, 0.92; 99.5% CI, 0.89-0.95; *P* < .001). There was also a significant decrease (40%; RR, 0.60; 99.5% CI, 0.47-0.77; *P* < .001) in ED charges after the intervention. The overall changes in the postintervention period were slightly higher for total charges, showing a 10% decrease in charges (RR, 0.90; 99.5% CI, 0.86-0.93; *P* < .001). Although the rate of change in ED charges of 12% was statistically significant after the first year of the intervention (RR, 0.88; 99.5% CI, 0.81-0.96; *P* < .001), the relative rate of change in charges was not as robust (RR, 0.66; 99.5% CI 0.38-1.16; *P* = .04). All other findings were robust and supported our main results.

**Table 3.** Level and Trends in ED Utilization: Segmented Regression of Interrupted Time-Series Analysis

Outcome measure	RR (99.5% CI)	*P* value
ED visits		
Level change^a^		
Pre-intervention vs after 3 y	0.61 (0.48-0.76)	<.001
After 1 y	0.66 (0.49-0.90)	<.001
After 2 y	0.61 (0.47-0.79)	<.001
After 3 y	0.60 (0.48-0.76)	<.001
Slope change^b^		
Pre-intervention vs after 3 y	0.92 (0.89-0.95)	<.001
After 1 y	0.90 (0.86-0.95)	<.001
After 2 y	0.92 (0.88-0.95)	<.001
After 3 y	0.92 (0.89-0.95)	<.001
ED charges		
Level change^a^		
Pre-intervention vs after 3 y	0.60 (0.47-0.77)	<.001
After 1 y	0.66 (0.38-1.16)	.04
After 2 y	0.60 (0.41-0.87)	<.001
After 3 y	0.60 (0.44-0.80)	<.001
Slope change^b^		
Pre-intervention vs after 3 y	0.90 (0.86-0.93)	<.001
After 1 y	0.88 (0.81-0.96)	<.001
After 2 y	0.90 (0.85-0.95)	<.001
After 3 y	0.90 (0.86-0.94)	<.001

Abbreviations: ED, emergency department; RR, relative risk.

^a^ Level change measures how much of the abrupt change in the measure (ED visits or charges) immediately after the intervention is associated with the intervention. Significant level change indicates that the change from pre–Healthy Outcome Plan to immediately post–Healthy Outcome Plan is associated with the HOP intervention.

^b^ Slope change (or trend change) measures the month-to-month change (movement) before or after the intervention. The significant slope change indicates the sustained association of the Healthy Outcome Plan intervention with changes in ED visits and charges from year 1 to 3.

**Figure 1.**
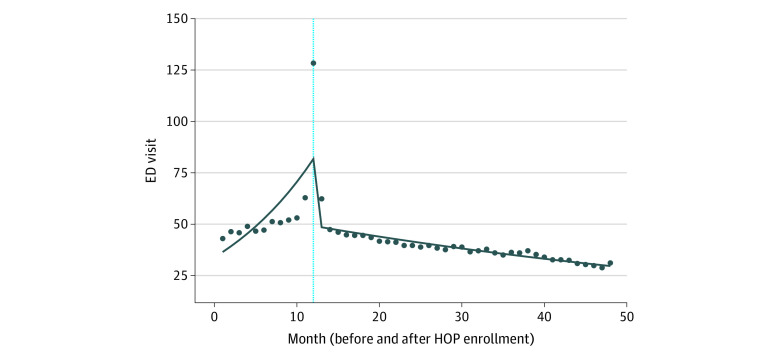
Segmented Regression of Interrupted Time-Series Analysis Before and After Healthy Outcomes Plan (HOP), Emergency Department (ED) Visits The vertical line indicates the implementation of the HOP.

**Figure 2.**
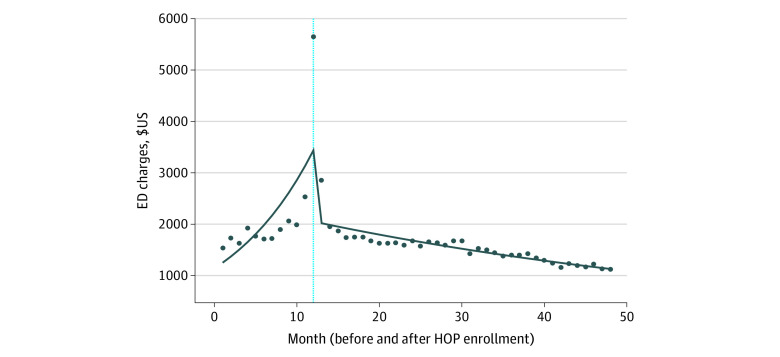
Segmented Regression of Interrupted Time-Series Analysis Before and After Healthy Outcomes Plan (HOP), Emergency Department (ED) Charges in US Dollars The vertical line indicates the implementation of the HOP program.

Trends in ED visits and charges before and after the intervention are shown in Figures 1 and 2, with the actual value of each measure over time by month and an estimated model fit. Reflective of the utilization enrollment criteria, there was a small but significant spike in visits and charges within the 1-month pre-intervention and postintervention periods, with a significant drop directly after the initiation of a sustained care plan. The sensitivity analysis restricting records at the time of enrollment did not alter the general trends for the interrupted time series models.

## Discussion

An estimated 13% to 27% of ED visits in the United States could be managed in physician offices.^14,15^ The SC HOP program has several interventions focused on lowering the utilization of acute care services. These initiatives extended complex care management to 31 166 uninsured individuals, leading to insurance coverage for many participants. Among HOP participants with at least 18 months of enrollment, we observed a 44% decrease in acute service use and an estimated 46% reduction in ED charges. The most dramatic changes were found among high-frequency utilizers and those with chronic physical and behavioral health conditions. Reductions in ED charges were sustained throughout the postintervention period, suggesting that improvements in health outcomes drove the cost reductions. This is consistent with previous HOP findings in both the ED and inpatient setting where higher rate reductions were observed among high-frequency users.^6^

Previous interventions to reduce ED utilization include linking uninsured patients to local primary care clinicians, community-based partnerships, case management, and personalized care plans.^1,2,3,4,5^ SC’s HOP maintains many of the mechanisms that may lead to a reduction in potentially preventable and modifiable health care use, including identifying and targeting patients with high health care needs and costs through a balanced approach; engaging these patients in managing their health; and obtaining health care professional buy-in.^16^ It is a state-based initiative with an essential component of chronic disease management that incentivizes hospitals to coordinate appointments for participants and increase access to primary care clinicians. It also integrates the Global Appraisal of Individual Needs–Short Screener to proactively screen for behavioral health needs, as well as the Patient Activation Measure, a measure of the ability to engage in self-care and medication management, which we found to be associated with improved outcomes.^17^ Like other ED-based and primary care–based models, we found clear associations between the HOP program and reductions in ED visits and charges, as well as other decreases in the type and severity of admissions across both rural and urban hospitals.^18^ We have also observed consistent findings for inpatient stays and charges in the larger program evaluation, resulting in more than cost neutrality when comparing the budget without DSH allotments vs the cost avoided.^6^ Reducing uncompensated care charges reduces strains on hospital budgets. These savings enable hospitals and health care systems to make new investments in equipment and technology, maintain capacity, and make closures less likely.

This evaluation is timely, given renewed national efforts by the US Centers for Medicare & Medicaid Services (CMS) to motivate health care professionals to collaborate in addressing social determinants of health.^19^ Current CMS guidance for state Medicaid directors encourages value-based strategies to improve population health and reduce costs by addressing social determinants of health.^20^ The SC HOP program could serve as a model for how other state Medicaid agencies can incentivize hospitals to partner with safety net hospitals to comprehensively meet the needs of patients to improve their clinical outcomes and, consequently, reduce cost.

Nationally, Medicaid expansion has played a role in decreasing the likelihood of frequent ED use.^19^ For the 10 states that have not expanded Medicaid, eligibility for adults remains limited, resulting in an estimated 1.9 million uninsured adults with low income falling into the coverage gap.^21^ The average income threshold is just 38% of the poverty rate, or $9446 for a family of 3, and is typically unavailable to persons without children.^22^ Many of these individuals are high users of acute care services and often fail to meet the eligibility for Medicaid enrollment due to its low poverty thresholds. These findings demonstrate the HOP model’s potential in other nonexpansion states, possibly through an 1115 demonstration waiver, where restricted Medicaid eligibility for adults still exists.

### Limitations

This study has several limitations. First, enrollment and utilization data were contingent on what was provided by participating hospitals and, therefore, could not be validated, resulting in potential measurement error. For example, data on final hospital payments, which are typically lower than initial charges, were not available for analysis. Although hospital charges are more universally interpretable, the total savings could be lower based on payments that were made. Second, while the interrupted time series analysis is a strong method for quasi-experimental study designs, it is important to acknowledge that factors outside hospital settings may influence the analysis. Study findings may not be generalizable, and fidelity of the model varied across individual sites, as observed by some participants not having the required ED visits prior to enrollment. Given the duration of the program and its enrollment parameters, sample attrition and regression to the mean were also considered. Our final sample did have higher acuity than the full sample, but mean reductions were observed across all 3 utilization groups. Third, consistent with the interrupted time series method, the data were aggregated at time point, not by individual-level utilization. As a result, unlike the GEE models, the interrupted time series analysis did not control for patient-level confounders, and we could not directly describe individual-level associations. Fourth, it should be noted that not all cohort participants had visits that would be categorized by the NYU algorithm. The frequencies for those visits would be lower than the total study population. Similarly, our analysis was limited to a portion of all HOP participants. This stemmed from recommendations to limit HOP evaluations to enrollment of at least 18 months as well as enrollment age and conditions requirements. Additionally, given the available data, an analysis of nonhospital utilization outcomes was not feasible; however, to address this limitation, in-depth qualitative interviews with HOP coordinators were conducted in March 2022 to retrospectively understand the program’s successes and challenges across implementation, fiscal and administrative structure, and partnership. Ongoing evaluation efforts are also focused on the impact of COVID-19 on the status of the program, recent enrollment patterns, and outcomes for currently active participants by varying levels of continuous enrollment.

## Conclusions

In this retrospective cohort study, we found that the SC HOP program was associated with reduced ED visits and charges for uninsured patients with high health care needs and costs through hospital-community partnerships by providing enhanced screening, care coordination, and access to primary care services. At the end of the 2022-2023 legislative session, the SC legislature voted to approve a budget to end the HOP program effective July 1, 2023. Despite the program’s end, these findings demonstrate HOP’s effectiveness and potential for state Medicaid agencies seeking innovative ways to address complex care coordination and the social determinants of health to reduce the strain on hospital EDs. The study provides a new framework for delivering services to uninsured individuals through an innovative funding model.

## Notes

Supplement.
Data Sharing Statement
