# Supplementary material for: Association of Hospital Incentive Care Management Partnerships for Uninsured Patients With Emergency Department Utilization
Source: JAMA Netw Open. 2023 Jul 11;6(7):e2322798. doi: 10.1001/jamanetworkopen.2023.22798 (PMC10336620; doi:10.1001/jamanetworkopen.2023.22798)
Supplement: Supplement. — Data Sharing Statement [file jamanetwopen-e2322798-s001.pdf]

Data Sharing Statement

Gareau. Association of Hospital Incentive Care Management Partnerships for Uninsured Patients With Emergency Department Utilization. *JAMA Netw Open*. Published July 11, 2023. doi:10.1001/jamanetworkopen.2023.22798

Data
Data available: No

Additional Information
Explanation for why data not available: The data is governed by a Data User Agreement allowing the use of this data solely for evaluation and publication. The IRB protocol obtains permission from participants for these two purposes precluding the release of this data.
